# Biological Control of Take-All and Growth Promotion in Wheat by *Pseudomonas chlororaphis* YB-10

**DOI:** 10.3390/pathogens10070903

**Published:** 2021-07-17

**Authors:** Wen Xu, Lingling Xu, Xiaoxu Deng, Paul H. Goodwin, Mingcong Xia, Jie Zhang, Qi Wang, Runhong Sun, Yamei Pan, Chao Wu, Lirong Yang

**Affiliations:** 1 Institute of Plant Protection Research, Henan Academy of Agricultural Sciences, Zhengzhou 450002, China; xuwen@hnagri.org.cn (W.X.); ddengxiaoxu@hnagri.org.cn (X.D.); xiamingcong@hnagri.org.cn (M.X.); zhangjie@hnagri.org.cn (J.Z.); sunrunhong@hnagri.org.cn (R.S.); panyamei@hnagri.org.cn (Y.P.); wuchao@hnagri.org.cn (C.W.); 2 Henan International Joint Laboratory of Crop Protection, Henan Biopesticide Engineering Research Center, Henan Academy of Agricultural Sciences, Zhengzhou 450002, China; 3 Department of Plant Pathology, College of Plant Protection, Henan Agricultural University, Zhengzhou 450002, China; xulingling@hnagri.org.cn; 4 School of Environmental Sciences, University of Guelph, Guelph, ON N1G2W1, Canada; pgoodwin@uoguelph.ca; 5 Department of Plant Pathology, College of Plant Protection, China Agricultural University, Beijing 100083, China; wangqi@cau.edu.cn

**Keywords:** take-all, biocontrol, growth-promoting, *Pseudomonas chlororaphis*, *Gaeumannomyces graminis* var. *tritici*

## Abstract

Wheat is a worldwide staple food crop, and take-all caused by *Gaeumannomyces graminis* var. *tritici* can lead to a tremendous decrease in wheat yield and quality. In this study, strain YB-10 was isolated from wheat rhizospheric soil and identified as *Pseudomonas chlororaphis* by morphology and 16S rRNA gene sequencing. *Pseudomonas chlororaphis* YB-10 had extracellular protease and cellulase activities and strongly inhibited the mycelium growth of *Gaeumannomyces graminis* var. *tritici* in dual cultures. Up to 87% efficacy of *Pseudomonas chlororaphis* YB-10 in controlling the take-all of seedlings was observed in pot experiments when wheat seed was coated with the bacterium. *Pseudomonas chlororaphis* YB-10 was also positive for indole acetic acid (IAA) and siderophore production, and coating wheat seed with the bacterium significantly promoted the growth of seedlings at 10^7^ and 10^8^ CFU/mL. Furthermore, treatment with *Pseudomonas chlororaphis* YB-10 increased activities of the wheat defense-related enzymes POD, SOD, CAT, PAL and PPO in seedlings, indicating induced resistance against pathogens. Overall, *Pseudomonas chlororaphis* YB-10 is a promising new seed-coating agent to both promote wheat growth and suppress take-all.

## 1. Introduction

Take-all caused by *Gaeumannomyces graminis* var. *tritici* (*Ggt*) is one of the most destructive root diseases of wheat worldwide and significantly reduces wheat grain yield after severe outbreaks [1,2,3]. The fungus *G. graminis* var. *tritici* damages wheat by rotting the root and base of the stem, disrupting the uptake and flow of water and nutrients, and eventually causing death of infected plants [2,4,5]. As with other soil-borne plant diseases, take-all is difficult to manage by current approaches such as the development of resistant cultivars, use of chemical pesticides, crop rotation and tillage [6,7,8,9,10]. However, several studies in the past thirty years have demonstrated that antagonistic bacteria and fungi can be applied to wheat to control take-all disease. This is considered to be a more efficient and ecologically friendly alternative management approach [4,11,12,13,14]. Therefore, the identification of effective antagonistic microorganisms to control take-all disease in wheat is an urgent need for sustainable wheat production.

Many studies have revealed that antagonistic bacteria not only play significant roles in controlling different plant diseases as biological control agents (BCAs), but can also increase plant growth as plant growth-promoting rhizobacteria (PGPR) [15,16,17,18,19]. Among them, isolates of *Pseudomonas* have proven to be capable of producing antibiotic compounds to suppress plant pathogens such as *G. graminis* var. *tritici*, and possess plant growth-promoting characteristics such as phosphate solubilization, siderophores, 1-aminocyclopropane-1-carboxylate (ACC) deaminase and indole-3-acetic acid (IAA), etc. [20,21,22,23,24]. For example, *P. chlororaphis* 30–84 produced the antibiotic sphenazine-1-carboxylic acid (PCA) that effectively inhibited take-all [23]; *P. fluorescens* 2–79 also produced the antibiotics PCA that strongly inhibited *G. graminis* var. *tritici* in culture [25]; and *P. fluorescens* VUPf5 produced siderophores, hydrogen cyanide, protease, phenazine and volatile metabolites that suppressed take-all disease by 85% in pots [26]. Furthermore, isolates of *Pseudomonas* have been shown to be both BCAs and PGPRs in other plants. For example, *P. aeruginosa* 2apa produced IAA, salicylic acid and siderophores, and promoted tomato growth along with antimicrobial activity against a wide range of pathogens [27]. *Pseudomonas aeruginosa* PN1 produced IAA, cyanogens, solubilized phosphorus, siderophore, chitinase and β-1, 3-glucanase and improved the growth of chirpine seedlings along with exhibiting a strong antagonistic property against *Macrophomina phaseolina* [17]. In addition, inoculations with different *Pseudomonas* strains have triggered defense-related enzymes in plants, such as peroxidase (POD), superoxide dismutase (SOD), catalase (CAT), phenylalanine ammonia lyase (PAL) and polyphenoloxidase (PPO), indicating that they can produce induced systemic resistance against pathogens [28,29,30,31,32].

In this study, strain YB-10 was isolated from wheat rhizospheric soil and identified as *Pseudomonas chlororaphis* by morphology and 16S rRNA gene sequencing. It exhibited strong antagonistic activity against *G. graminis* var. *tritici* in dual cultures and was shown to have a number of potential growth-promoting and biocontrol traits. When used to coat wheat seeds, *P. chlororaphis* YB-10 not only promoted the growth of wheat seedlings, but also suppressed take-all caused by *G. graminis* var. *tritici*. These results demonstrated that *P. chlororaphis* YB-10 could be a promising BCA of wheat and PGPR against take-all disease.

## 2. Materials and Methods

### 2.1. Isolation of StrainYB-10

Soil sample collection and isolation of strain YB-10 from wheat rhizosphere used in this study were as previously described in [12].

### 2.2. Identification of Strain YB-10

A single colony of strain YB-10 was inoculated in Nutrient Broth and grown for 24 h at 30 °C, shaking at 150 rpm. Genomic DNA of YB-10 was extracted by the MiniBEST Bacterial Genomic DNA Extraction Kit Ver. 3.0 (TaKaRa, Beijing, China) following the manufacturer’s instructions. The 16S rRNA gene fragment was amplified with primers27F (5′-AGAGTTTGATCCTGGCTCAG-3′) and 1492R (5′-GGTTACCTTGTTACGACTT-3′). A phylogenetic tree of the 16S rRNA gene sequences was constructed with MEGA 7.0 using the neighbor joining method [33].

### 2.3. Detection of PGP and Antifungal Traits

IAA production was detected with the Salkowski method as described by Glickmann and Dessaux [34]. 

Siderophore production was determined on Chrome Azurol S blue agar (CAS) [35]. A single colony of *P. chlororaphis* YB-10 was inoculated on CAS and cultured at 30 °C in the dark. Siderophore production was indicated by the color change from blue to orange around the colonies. 

A single colony of *P. chlororaphis* YB-10 was inoculated onto carboxymethylcellulose agar and incubated for 7 days at 30 °C. The plates were flooded with 1% (*m*/*v*) Congo red and then washed with distilled water. The cellulose activity was detected as clear zones around the colonies [36]. 

Protease activity was detected on skim milk agar (5 g NaCl, 0.1 g CaCl_2_, 10 g peptone, 10 g skim milk and 18 g of agar per liter (pH 7.2)). A single colony of *P. chlororaphis* YB-10 was placed on the medium and incubated at 30 °C for 5 days. Protease activity of *P. chlororaphis* YB-10 was observed by the clear zones around the colonies [37].

### 2.4. Plant Growth Promotion Assay of P. chlororaphis YB-10 on Wheat Seedlings

*P. chlororaphis* YB-10 was cultured in 100 mL NB broth for 36 h at 30 °C with shaking at 180 rpm. Wheat (*Triticum aestivum* L.) seeds of cultivar Zhengmai 366 were surface-sterilized in 75% ethanol (*v*/*v*) for 30s and then rinsed with sterile water three times. Wheat seeds were air-dried and then soaked with a suspension of *P. chlororaphis* YB-10 at 10^7^ CFU/mL, 10^8^ CFU/mL or 10^9^ CFU/mL for 2 h. The seeds were then placed in a Petri dish with a diameter of 9 cm in the greenhouse at 25 °C with a 12 h light/12 h dark photoperiod for 10 days. Each treatment had three replicates. Seeds soaked with NB medium were used as control.

### 2.5. Biocontrol Assay of P. chlororaphis YB-10 Against Take-All inWheat

Wheat seeds were soaked with *P. chlororaphis* YB-10 at 10^7^~10^8^ CFU/mL or sterile NB medium as described above. The seeds were air-dried, and 15 seeds were planted per pot (10 cm high, 10 cm diameter) filled with 350 g potting soil. The pots were placed in the greenhouse at 25 °C. *Gaeumannomyces graminis* var. *tritici* GGT-007, isolated from Xiping, Henan Province in 2014, that was previously shown to be highly virulent to wheat (data not shown), was grown on PDA for 10 days at 25 °C. A 5 mm disc of mycelium with agar from the growing edge of the colony was placed at a 1cm depth in a pot and then a wheat seed was attached to the surface of the mycelial disc. This method of inoculation was shown to achieve a 100% infection rate [12]. Four treatments were made:

1. Seeds soaked with *P. chlororaphis* YB-10 and not inoculated with *Ggt*;
2. Seeds soaked with *P. chlororaphis* YB-10 and inoculated with *Ggt*;
3. Seeds not soaked with *P. chlororaphis* YB-10 and not inoculated with *Ggt*;
4. Seeds not soaked with *P. chlororaphis* YB-10 and inoculated with *Ggt*.

At 3 weeks post-planting, the treated wheat seedlings were removed from pots and roots were washed free of soil. The symptoms of take-all, disease index and control efficacy were measured as per Yang et al [12]. The experiment was replicated three times.

### 2.6. Activities of Defense Enzymes in Wheat Seedlings

At 3 weeks post-planting, the shoots of the treated wheat seedlings were harvested and stored at −80 ℃. In brief, 0.5 g shoots were ground in liquid nitrogen, and a 1 mL extraction buffer was added. The buffer was then centrifuged, and the supernatant was removed and used for enzyme assays. The activities of SOD, POD, CAT, PAL and PPO were measured following the procedures of the manufacturer (Solarbio, Beijing, China) using an SOD assay kit (Cat. No. BC0175), POD assay kit (Cat. No. BC0095), CAT assay kit (Cat. No. BC0205), PAL assay kit (Cat. No. BC0215) and PPO assay kit (Cat. No. BC0195), respectively. Absorbance was determined by a plate reader (Tecan Spark, Tecan, Switzerland). 

### 2.7. Statistical Analysis

Statistical analysis was performed using SPSS v21.0, one-way analysis of variance (ANOVA). Means were compared with Duncan’s multiple range tests at a probability of *p* ≤ 0.05.

## 3. Results

### 3.1. Screening and Identification of Strain YB-10 

**Figure 1:**
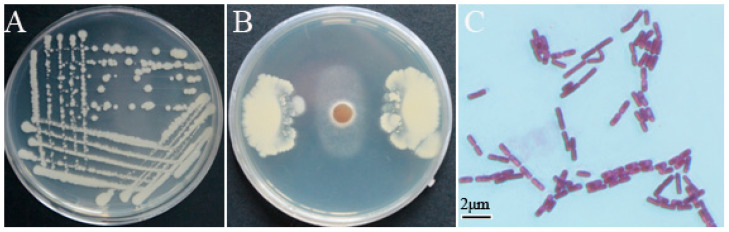
Characteristics of *Pseudomonas chlororaphis* YB-10. (**A**) Morphology of *P. chlororaphis* YB-10 on NA. (**B**) Inhibition of growth of *G. graminis* var. *tritici* by *P. chlororaphis* YB-10. (**C**) Gram staining of *P. chlororaphis* YB-10.

**Figure 2:**
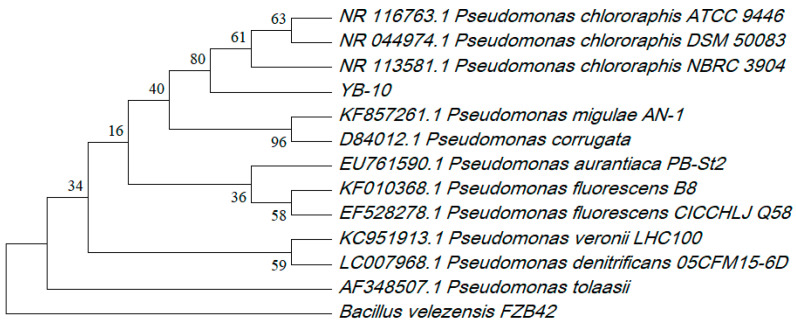
Phylogenetic tree of *P. chlororaphis* YB-10 and 12 other bacteria isolates based on 16S rDNA sequences. Bootstrap values were from 1000 replicates.

Strain YB-10 isolated from wheat rhizospheric soil was milky-white, non-pigmented on NA and Gram-negative (Figure 1A,C). Dual cultures of strain YB-10 and *G. graminis* var. *tritici* on NA displayed strong growth inhibition of *G. graminis* var. *tritici* by YB-10 (Figure 1B). A search using the 16S rDNA sequence of YB-10 with the Basic Local Alignment Search Tool (BLAST) against the NCBI nr database demonstrated that strain YB-10 had 100% sequence homology to *P. chlororaphis*. The sequence data were submitted to GenBank with the accession number MW093066. The phylogenetic tree of the 16S rRNA gene sequences revealed that strain YB-10 was in a cluster of *P. chlororaphis* isolates (Figure 2).

### 3.2. Characterization of In Vitro Antifungal and PGP Traits of P. chlororaphis YB-10

**Figure 3:**
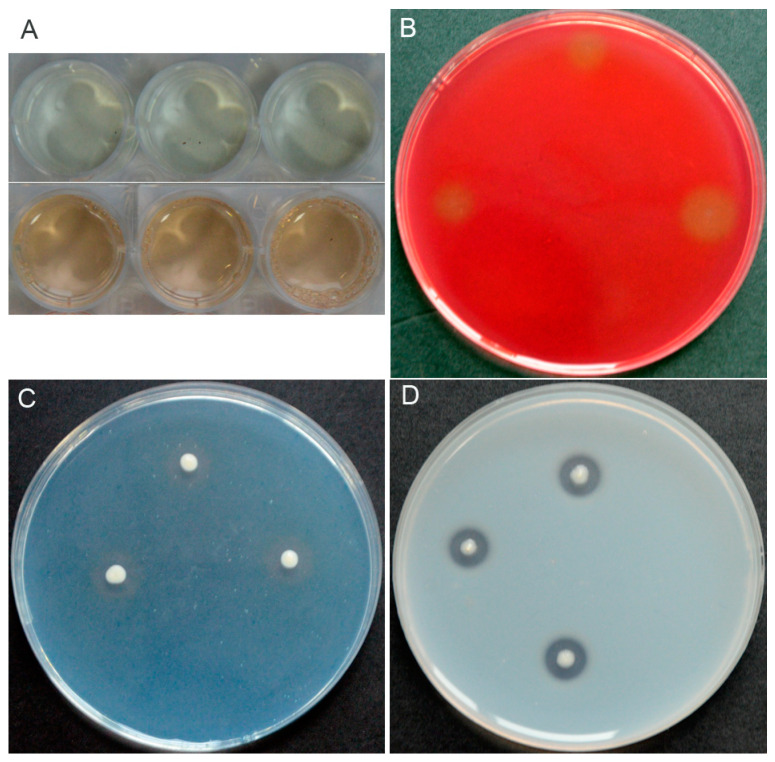
In vitro antifungal and PGP traits of *P. chlororaphis* YB-10. (**A**) Pink coloration indicating IAA production. (**B**) Clear zones indicating cellulase activity. (**C**) Orange halos indicating siderophore production. (**D**) Clear zones indicating protease activity.

*P. chlororaphis* YB-10 was capable of synthesizing indole acetic acid (IAA) (Figure 3A), producing siderophores (Figure 3C), and secreting extracellular cellulase and protease (Figure 3B,D). However, *P. chlororaphis* YB-10 showed no phosphate solubilization or 1-aminocyclopropane-1-carboxylic acid (ACC) deaminase activities (data not shown). 

### 3.3. Effect of P. chlororaphis YB-10 Against Take-All in Wheat Seedlings

**Figure 4:**
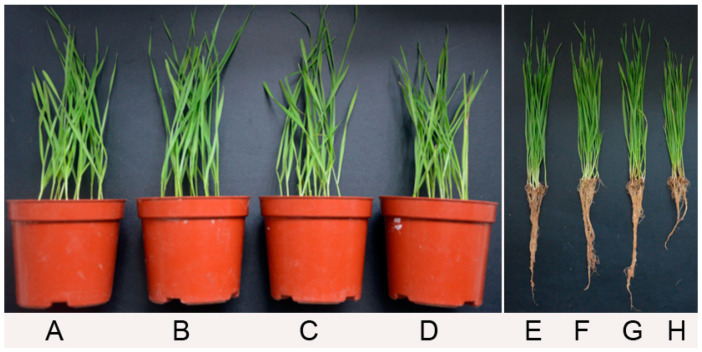
Effect of *P. chlororaphis* YB-10 against take-all in wheat seedlings. (**A**,**E**) Wheat seeds treatments of *P. chlororaphis* YB-10 without *G. graminis* var. *tritici* inoculation. (**B**,**F**) Wheat seeds treatments of *P. chlororaphis* YB-10 with *G. graminis* var. *tritici* inoculation. (**C**,**G**) Control treatments without *G. graminis* var. *tritici* inoculation. (**D**,**H**) Control treatments with *G. graminis* var. *tritici* inoculation.

For wheat seedlings at 21 days after *G. graminis* var. *tritici* inoculation without seed treatment of *P. chlororaphis* YB-10 in pots under greenhouse conditions (Figure 4), symptoms of take-all, including blackened or chocolate brown lesions on the stembase and root, were observed (Figure 4D,H). Limited typical disease symptoms were observed in wheat seedlings without *G. graminis* var. *tritici* inoculation and without seed treatment of *P. chlororaphis* YB-10, indicating a low level of *G. graminis* var. *tritici* in the potting soil (Figure 4C,G). However, no typical disease symptoms were observed with or without *G. graminis* var. *tritici* inoculation with wheat seed treatment of *P. chlororaphis* YB-10 (Figure 4A,E,B,F, respectively).The disease index of wheat seedlings was 7.14 ± 0.11 and 6.67 ± 0.28 with seed treatment of *P. chlororaphis* YB-10 without or with *G. graminis* var. *tritici* inoculation, respectively, but the disease index was 78.57 ± 0.47 and 51.59 ± 1.11 without seed treatment of *P. chlororaphis* YB-10 with or without *G. graminis* var. *tritici* inoculation, respectively (Table 1). Disease incidence with seed treatment of *P. chlororaphis* YB-10 with or without *G. graminis* var. *tritici* inoculation was only 0.07%. However, disease incidence was 92.86% ± 0.62% and 64.29% ± 0.72% without seed treatment of *P. chlororaphis* YB-10 with or without *G. graminis* var. *tritici* inoculation, respectively. The take-all control efficacy in wheat seedlings was 86–87% with seed treatment of *P. chlororaphis* YB-10.

### 3.4. Growth Promotion of Wheat Seedlings after P. chlororaphis YB-10 Inoculation

**Figure 5:**
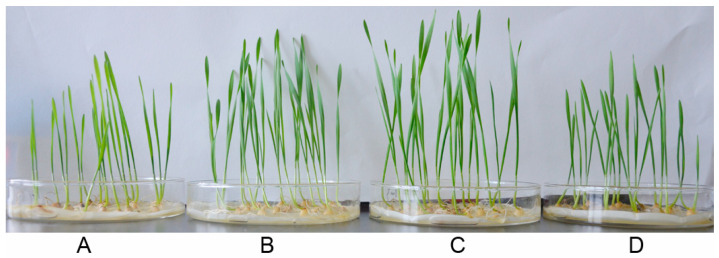
Growth promotion of wheat seedlings by *P. chlororaphis* YB-10. (**A**) Wheat seeds soaked without *P. chlororaphis* YB-10. (**B**) Wheat seeds soaked with *P. chlororaphis* YB-10 at 10^7^ CFU/mL. (**C**) Wheat seeds soaked with *P. chlororaphis* YB-10 at 10^8^ CFU/mL. (**D**) Wheat seeds soaked with *P. chlororaphis* YB-10 at 10^9^ CFU/mL.

For wheat seeds soaked with *P. chlororaphis* YB-10 at different doses, *P. chlororaphis* YB-10 at 10^7^ and 10^8^ CFU/mL significantly promoted the growth of wheat seedlings, but *P. chlororaphis* YB-10 at 10^9^ CFU/mL had an inhibitory effect on the growth of wheat seedlings (Figure 5). The fresh weights of shoots of wheat seedlings inoculated with *P. chlororaphis* YB-10 at 10^7^ and 10^8^ CFU/mL were 1.68 ± 0.06 g and 1.72 ± 0.04 g, respectively, compared to 1.18 ± 0.07 g without inoculation with *P. chlororaphis* YB-10. The fresh weights of roots of wheat seedlings inoculated with *P. chlororaphis* YB-10 at 10^7^ and 10^8^ CFU/mL were 1.53 ± 0.03 g and 1.51 ± 0.05 g, respectively, compared to 0.91 ± 0.02 g without inoculation with *P. chlororaphis* YB-10. The lengths of shoots of wheat seedlings inoculated with *P. chlororaphis* YB-10 at 10^7^ and 10^8^ CFU/mL were 12.41 ± 0.52 cm and 12.43 ± 0.67 cm, respectively, compared to 8.21 ± 0.42 cm without inoculation with *P. chlororaphis* YB-10. Overall, seed treatment with *P. chlororaphis* YB-10 at 10^7^ and 10^8^ CFU/mL caused a significant improvement (*p* < 0.05) in fresh weights of shoots and roots and in the lengths of shoots of wheat seedlings, but seed treatment with *P. chlororaphis* YB-10 at 10^9^ CFU/mL did not differ significantly (*p* > 0.05) compared with non-inoculated treatment (Table 2).

### 3.5. Effect of P. chlororaphis YB-10 on Activities of Defense Enzymes in Wheat Seedlings

**Figure 6:**
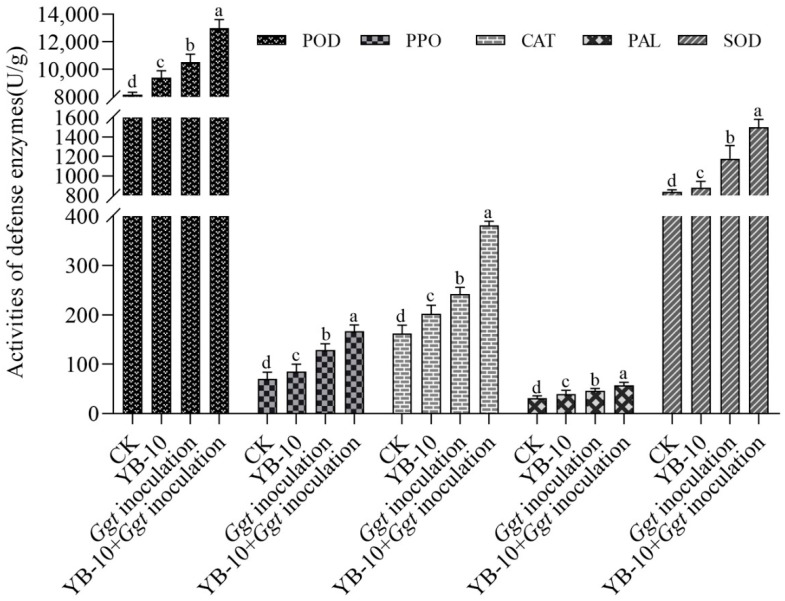
Five defense enzymes activities of wheat shoots under different treatments. peroxidase (POD), superoxide dismutase (SOD), catalase (CAT), phenylalanine ammonia lyase (PAL) and polyphenoloxidase (PPO). Data are mean ± standard deviation (SD) of three replicates; Different letters (a–d) in the same enzyme indicate significant difference at *p* values < 0.05 level.

POD activity of wheat shoots was 9406.51 ± 200.20 U/g and 12,925.14 ± 998.21 U/g with seed treatment of *P. chlororaphis* YB-10 without or with *G. graminis* var. *tritici* inoculation, respectively, whereas the POD activity was 7261.47 ± 251.01 U/g and 10,517.07 ± 234.12 U/g without seed treatment of *P. chlororaphis* YB-10 without or with *G. graminis* var. *tritici* inoculation, respectively (Figure 6). PPO activity of wheat shoots was 85.46 ± 6.16 U/g and 166.95 ± 4.87 U/g with seed treatment of *P. chlororaphis* YB-10 without or with *G. graminis* var. *tritici* inoculation, respectively, whereas PPO activity was 70.16 ± 5.18 U/g and 128.78 ± 5.67 U/g without seed treatment of *P. chlororaphis* YB-10 without or with *G. graminis* var. *tritici* inoculation, respectively. CAT activity of wheat shoots was 201.73 ± 7.18 U/g and 387.59 ± 10.94 U/g in seed treatment of *P. chlororaphis* YB-10 without or with *G. graminis* var. *tritici* inoculation, respectively, whereas CAT activity was 161.97 ± 7.40 U/g and 241.73 ± 5.76 U/g without seed treatment of *P. chlororaphis* YB-10 without or with *G. graminis* var. *tritici* inoculation, respectively. PAL activity of wheat shoots was 39.78 ± 3.46 U/g and 57.38 ± 4.11 U/g with seed treatment of *P. chlororaphis* YB-10 without or with *G. graminis* var. *tritici* inoculation, respectively, whereas PAL activity was 30.87 ± 2.33 U/g and 45.61 ± 2.47 U/g without seed treatment of *P. chlororaphis* YB-10 without or with *G. graminis* var. *tritici* inoculation, respectively. SOD activity of wheat shoots was 881.57 ± 25.97 U/g and 1514.12 ± 54.61 U/g with seed treatment of *P. chlororaphis* YB-10 without or with *G. graminis* var. *tritici* inoculation, respectively, whereas SOD activity was 748.87 ± 16.23 U/g and 1178.64 ± 62.32 U/g without seed treatment of *P. chlororaphis* YB-10 without or with *G. graminis* var. *tritici* inoculation, respectively. In general, wheat seeds soaked with *P. chlororaphis* YB-10 resulted in significant increases in POD, SOD, CAT, PAL and PPO activities of wheat shoots regardless of *G. graminis* var. *tritici* inoculation. 

## 4. Discussion

In recent years, many studies have demonstrated that *Pseudomonas* strains from the plant rhizosphere could control diseases caused by a variety of pathogens [21,38,39,40]. For example, *Pseudomonas fluorescens* CV6 had broad antimicrobial activity against 12 pathogens [41], and *P. fluorescens* VUPf5 strongly inhibited *G. graminis* var. *tritici* in vitro and greenhouse, suppressing take-all disease by 85% [26]. Many of these strains have antifungal attributes, such as β-1,3-glucanase, cellulase, protease and chitinase activities [42,43]. It is also well known that a number of *Pseudomonas* strains from the plant rhizosphere can promote plant growth [18,20,40,41]. These strains show multiple growth-promoting properties, including IAA production, siderophore production, hydrogen cyanide production and phosphate solubilization [42,43]. Further screening of new *Pseudomonas* isolates is desirable for creating new options for controlling plant diseases and promoting plant growth in sustainable agriculture systems. 

In this study, *P. chlororaphis* strain YB-10 was isolated from wheat rhizosphere, which had cellulose and protease activities and suppressed take-all caused by *G. graminis* var. *tritici* with very high efficacy. In addition, *P. chlororaphis* YB-10 was positive for IAA and siderophore production and significantly promoted the growth of wheat seedlings at 10^7^ and 10^8^ CFU/mL. However, the growth promotion effect was dose-dependent as wheat seedlings soaked with *P. chlororaphis* YB-10 at 10^9^ CFU/mL did not differ significantly compared with non-inoculated treatment. Therefore, *P. chlororaphis* YB-10 showed itself to be an effective PGPR for wheat seedlings and BCA against take-all of wheat.

In nature, plants are attacked by various pathogens inducing the production of different defense-related enzymes that play important roles in disease resistance [44,45]. The level of defense-related enzymes, such as POD, PPO, CAT, PAL and SOD, have been correlated with plant disease resistance [46,47]. Many studies have reported that biocontrol agents such as *P. fluorescens*, *Pseudomonas* sp. 23S, *Bacillus subtilis*, *P. chlororaphis* ToZa7 and *Clonostachys rosea* IK726 induced the expression of defense-related enzymes including SOD, CAT, PAL and PPO [24,31,46,48,49]. In this study, the activities of POD, PPO, CAT, PAL and SOD were increased significantly with seed treatment of *P. chlororaphis* YB-10 in healthy plants (i.e., without *G. graminis* var. *tritici* inoculation); activities were also increased with *G. graminis* var. *tritici* inoculation alone, indicating that the enzymes can be induced by the bacterium or pathogen alone. However, the highest levels detected were with seed treatment of *P. chlororaphis* YB-10 with *G. graminis* var. *tritici* inoculation, indicating increased levels of induction compared to those caused by *G. graminis* var. *tritici* infection. However, the promotion of plant growth by *P. chlororaphis* YB-10 indicates that there are multiple effects on the plants, and some of those other effects could have contributed to controlling take-all disease.

In summary, this is the first report that the wheat rhizosphere bacterium, *P. chlororaphis*, can both promote wheat growth and control take-all disease. Strain YB-10 was positive for IAA and siderophore production and had extracellular protease and cellulase activities, all of which could contribute to its beneficial effects. Moreover, seed treatment with *P. chlororaphis* YB-10 could later increase activities of defense-related enzymes in shoots. This indicates that the control of take-all by *P. chlororaphis* YB-10 could be related to induced systemic resistance (ISR) in the wheat seedlings. ISR has previously been shown to occur during the control of wheat take-all using treatments with other rhizobacterial species, and it is believed that ISR is the main mechanism for their ability to suppress take-all disease [50].

## Figures and Tables

**Table 1:** Disease incidence, disease index and control efficacy of *P. chlororaphis* YB-10 against take-all of wheat.

Treatment	Disease Incidence (%)	Disease Index	Control Efficacy (%)
CK	64.29 ± 0.72 ^b^	51.59 ± 1.11 ^b^	
YB-10	0.07 ± 0.00 ^c^	6.67 ± 0.28 ^c^	87.07 ± 0.54 ^a^
*Ggt* inoculation	92.86 ± 0.62 ^a^	78.57 ± 0.47 ^a^	
YB-10 + *Ggt* inoculation	0.07 ± 0.00 ^c^	7.14 ± 0.11 ^c^	86.16 ± 2.23 ^a^

Note: data are the Mean ± Standard deviation (SD); Different letters (a–c) in the same column indicate significant difference at *p* values < 0.05 level.

**Table 2:** Root and shoot fresh weights and shoot lengths of wheat seedlings by *P. chlororaphis* YB-10.

Treatment	Root Fresh Weight (g)	Shoot Fresh Weight (g)	Shoot Length (cm)
CK	0.91 ± 0.02 ^b^	1.18 ± 0.07 ^b^	8.21 ± 0.42 ^b^
YB-10(10^9^ CFU/mL)	0.89 ± 0.03 ^b^	1.17 ± 0.01 ^b^	8.255 ± 0.37 ^b^
YB-10(10^8^ CFU/mL)	1.51 ± 0.05 ^a^	1.72 ± 0.04 ^a^	12.43 ± 0.67 ^a^
YB-10(10^7^ CFU/mL)	1.53 ± 0.03 ^a^	1.68 ± 0.06 ^a^	12.41 ± 0.52 ^a^

Note: data are Mean ± SD; Different letters (a,b) in the same column indicate significant difference at *p* values < 0.05 level.

## Data Availability

Not applicable.
